# Filtering for Compound Heterozygous Sequence Variants in Non-Consanguineous Pedigrees

**DOI:** 10.1371/journal.pone.0070151

**Published:** 2013-08-05

**Authors:** Tom Kamphans, Peggy Sabri, Na Zhu, Verena Heinrich, Stefan Mundlos, Peter N. Robinson, Dmitri Parkhomchuk, Peter M. Krawitz

**Affiliations:** 1 Smart Algos, Berlin, Germany; 2 Institute for Medical Genetics and Human Genetics, Charité Universtätsmedizin, Berlin, Germany; 3 Max Planck Institute for Molecular Genetics, Berlin, Germany; University of Southern California, United States of America

## Abstract

The identification of disease-causing mutations in next-generation sequencing (NGS) data requires efficient filtering techniques. In patients with rare recessive diseases, compound heterozygosity of pathogenic mutations is the most likely inheritance model if the parents are non-consanguineous. We developed a web-based compound heterozygous filter that is suited for data from NGS projects and that is easy to use for non-bioinformaticians. We analyzed the power of compound heterozygous mutation filtering by deriving background distributions for healthy individuals from different ethnicities and studied the effectiveness in trios as well as more complex pedigree structures. While usually more then 30 genes harbor potential compound heterozygotes in single exomes, this number can be markedly reduced with every additional member of the pedigree that is included in the analysis. In a real data set with exomes of four family members, two sisters affected by Mabry syndrome and their healthy parents, the disease-causing gene *PIGO*, which harbors the pathogenic compound heterozygous variants, could be readily identified. Compound heterozygous filtering is an efficient means to reduce the number of candidate mutations in studies aiming at identifying recessive disease genes in non-consanguineous families. A web-server is provided to make this filtering strategy available at www.gene-talk.de.

## Background

In recessive genetic disorders, both copies of a certain gene are defective. For autosomal recessive genes, this means that the maternally as well as the paternally transmitted copy of a gene harbors a pathogenic mutation. The occurrence of a pathogenic mutation can be viewed as a random process, and many different pathogenic mutations have arisen for recessive genes in the human population over time. This also means the lower the kinship of the parents the higher is the chance that two different mutant alleles of the disease gene are present in a patient affected by a recessive disease, whereas in a closely related, consanguineous partnership it is more likely that an affected child will inherit the same pathogenic allele from both parents and thus be homozygous for the disease causing mutation. This translates to a simple rule of thumb: If the parents are non-consanguineous, the most likely explanation for a recessive disease is compound heterozygosity for two different pathogenic mutations. Exceptions from this rule of thumb may be founder mutations in certain populations and specific gain of function mutations in certain genes such as e.g. *FGFR2*.

A challenge in filtering sequence variants for compound heterozygotes is that one has to figure out whether the two heterozygous variants affect different copies of a gene or the same copy. Usually, that cannot be determined from a single DNA sequence, if the read length is less than the distance between the variants or if it is not possible to phase the haplotypes by any other means. However, when sequence variants of more than one family member are available, one can exclude certain variants based on rules of Mendelian inheritance. We will describe a set of rules that we used for compound heterozygous filtering and analyze how effectively the sequence variants can be reduced in certain case scenarios.

## Methods

We implemented a compound heterozygous filter in Ruby inside the GeneTalk framework [1]. We assume that the phenotype is fully penetrant and that all sequenced individuals are either affected or not affected. The first two rules work on a single variant level:

1 A variant has to be in a heterozygous state in all affected individuals.
2 A variant must not occur in a homozygous state in any of the unaffected individuals.

If a variant were homozygous in an unaffected individual it could not be disease causing, otherwise the individual would have to be affected, as both copies of the gene harbor the same mutation.

If the genotypes of both parents of an affected child are available and they are both unaffected there is a third rule that is very powerful in reducing the variants:

3 A variant that is heterozygous in an affected child must be heterozygous in exactly one of the parents.

This rule is a compact version of:

3a The variant must not be heterozygous in both parents.
3b The variant must be present in at least one of the parents.

**Figure 1:**
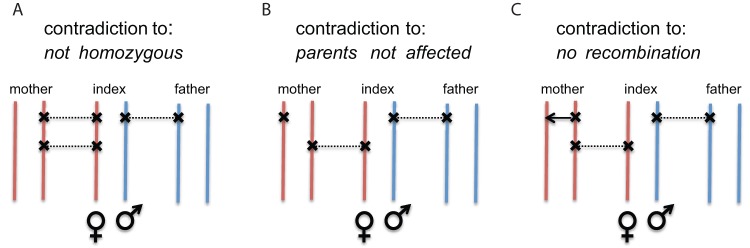
Compound Heterozygote Filtering Rules. If both parents of the index patient are unaffected it is not possible that one of the heterozygous disease causing mutations is present in a heterozygous state in both parents unless a recombination occurred between this variant and the second compound heterozygous mutation.

Rule 3a is applicable only if no recombination occurred between the tested loci in any of the parents. However, most genes have an extension considerably less than one megabase in the genome and thus the probability of a recombination is usually far below one per cent and the assumption of no recombination is well justified. In Figure 1, we illustrate why a variant that does not fulfill 3a may be removed as not pathogenic. If we keep such a variant it will result in a violation of one of prerequisites for a compound heterozygous mode of inheritance. Without loss of generality we may consider two heterozygous variants in an affected individual. One of them is in a heterozygous state in both unaffected parents. If the variant, for which mother and father are heterozygous, is transmitted by both of them to the index patient, then the variant would be homozygous in the child and would be therefore removed based on the first rule (Figure 1 A). The index patient could be heterozygous for both variants if both variants occurred on different copies of the gene in one of the parents. However, in this case this particular parent has to be affected as well and the third rule does not apply, as its assumption is not fulfilled (Figure 1B). The third case describes a scenario in which one of the parents has the two heterozygous mutations on the same copy of the gene, while the other parent is heterozygous for only one of them. In this scenario the child could be compound heterozygous only if a recombination happened in the germline of its ancestor (Figure 1C). As already mentioned, this case is so unlikely that we exclude it.

Rule 3b is applicable only if we assume that no *de novo* mutations occurred. The number of *de novo* mutations is estimated to be below five per exome per generation [2], [3], thus, the likelihood that an individual is compound heterozygous and at least one of these mutations arose *de novo* is low. If more than one family member is affected, *de novo* mutations are even orders of magnitudes less likely as a recessive disease cause. On the other hand, excluding these variants from the further analysis helps to remove many sequencing artifacts. In linkage analysis for example it is common practice of data cleaning to exclude variants as Mendelian errors if they cannot be explained by Mendelian inheritance.

When the filtering rules 1–3 have been applied on a single variant level, the fourth and fifth rule test on a gene level, whether enough variants remained to fulfill the requirements for a compound heterozygous mode of transmission and whether they were transmitted biparentally:

4 A gene must have two or more heterozygous variants in each of the affected individuals.
5 There must be at least one variant transmitted from the paternal side and one transmitted from the maternal side.

We did not use two as an upper bound in these rules as not necessarily all of the variants that pass these rules have to be pathogenic. However, as we will discuss later, a gene with many variants passing all five rules, is less likely to be a disease gene. The fifth rule makes sure that only genes pass the filter for which there are at least two heterozygous variants in the affected individuals that are transmitted in a biparental mode. Another way of phrasing this rule is: There must not be two identical haplotypes around the disease gene in an unaffected and an affected individual. If all heterozygous mutations of a gene in an affected individual match all the heterozygous mutations of the same gene in an unaffected individual of the family, then we exclude this gene. Imagine a scenario where sequence variants of only one grandfather or grandmother are available as unaffected control but not directly the sequence variants of the parents of the affected child. Let us assume that this index patient has exactly two heterozygous variants in a gene that match the genotypes of this gene in the grandmother. Excluding recombination this means that one of the parents of the child was either carrier of these two heterozygous variants or not. However, this means that one of these variants cannot be disease causing or rule three would be broken. The intervals for which we counted and compared the heterozygous genotypes were defined by the gene start and end points that we derived from Ensembl/BioMart [4]. All filters that we applied prior to the compound heterozygote filter are available through the GeneTalk platform. The allele frequency filter is based on genetic variation data from the 1000 genomes project [5] as well as the 5000 exomes project [6]. The effect of the variants on the protein level which is subject to the functional filter was predicted by ANNOVAR [7].

The length of a gene, as well as the variability of its sequence in a healthy reference population affects the probability to observe rare, heterozygous variants in a test individual. We derived the length of the coding sequence of the longest transcript per gene 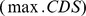 and determined the mean number of rare, heterozygous variant calls in healthy individuals 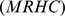 based on the 5000 exomes data [8] to assist in the interpretation of candidate genes after filtering. The 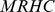 was computed by adding the frequencies of heterozygous variant calls for all positions in a gene that were below 0.01:
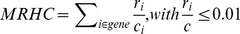
where *c_i_* is the total genotype count at position *i* and *r_i_* is the heterozygous genotype count at position *i*.

## Results

We tested the effectiveness of the compound heterozygotes filter in GeneTalk for single samples as well as in more complex pedigree structures. If single individuals are analyzed only rules 1 and 4 can be used. Rules 2 and 5 require data of at least one additional unaffected family member and rule 3 is applicable only if sequence data of both parents is available. Any additional filter settings should be used prior to rule 4 as this rule is more effective in reducing the number of candidate genes the lower the expected number of heterozygous variants is per gene. We used an allele frequency cutoff of 1% for heterozygous variants, removed all synonymous variants and known sequencing artifacts before applying the compound heterozygous filter. We choose the frequency cutoff of 1% as an upper bound, as this is above the allele frequency of the most common pathogenic alleles in cystic fibrosis (MIM 219700), one of the more common autosomal recessive disorders.

**Figure 2:**
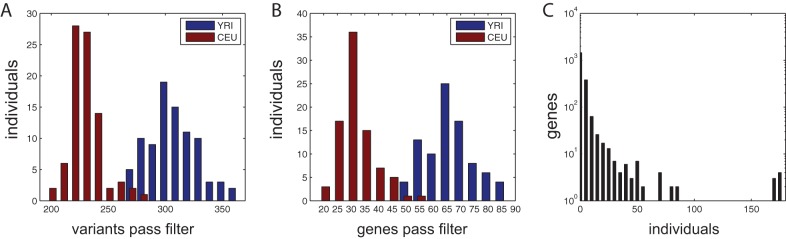
Exomes of 85 European individuals (CEU) as well as 88 African individuals (YRI) were filtered for rare compound heterozygous candidate variants. A) In average around 230 variants pass the filter in CEU exomes and 309 in YRI exomes. B) The potential compound heterozygotes are distributed over 31 genes in CEU individuals and 67 genes in YRI individuals. C) Altogether 1998 genes harbored potential compound heterozygous variants in the tested individuals and compound heterozygotes in 1066 genes occurred only in singular cases.

With these parameter settings, we filtered 85 European exomes (CEU) as well as 88 African exomes (YRI) available from the 1000 genomes project [5]. All these individuals are healthy and the numbers of variants as well as genes passing our filter settings serve as a background distribution that one has to expect when filtering single exome data. In the CEU exomes in average 230 variants distributed over 31 genes passed the filter, whereas in average 309 variants distributed over 67 genes passed the filter in the Yorubian exomes (Figure 2A and 2B). In the 173 tested individuals we identified variants as possible compound heterozygotes in altogether 1998 genes, and 1066 of these genes were unique to only one of the tested samples (Figure 2C).

As all these exomes were of healthy individuals it would be difficult to identify a single additional gene that passes the filters in a patient with a rare recessive disorder due to the true disease causing compound heterozygotes.

We then analyzed the effectiveness of the compound heterozygous filter for cases in which more than only a single exome of a family is available. For this purpose we used two parent-child trios of European (NA12878, NA12891, NA12892) as well as Yoruban (NA19238, NA19239, NA19240) descent from the 1000 genomes project. We assigned the status of the affected index to the offspring (NA12878, NA19238), so that for both trios all five rules were applicable. As before, in advance to the compound heterozygous filter, we reduced the exome variants of the trios to rare variants with a presumable effect on the protein level and removed known calling artifacts. This prior filtering step yielded 1668 variants in the European trio and 2653 variants in the Yoruban trio. The compound heterozygous filter reduced this number down to 48 variants in 17 genes in the European individual NA12878, while 68 variants in 29 genes passed in the Yoruban individual NA19238. If we remove genes for which compound heterozygous variants also occurred in other unrelated individuals (Figure 1C and Table S1), only twelve candidate genes remained in NA12878 *(CACHD1, DPP4, CACNA1D, PLXNA1, MECOM, PSMD2, TBC1D4, DCAF5, ZNF614, MYH9, PKDREJ, MAGEC1)* and in NA19238 *(CD180, ERAP2, EPB41L2, COL1A2, MUC16, AASS, PZP, ZMYM2, ZNF423, LRRC48, DMC1, GPR64)*. Thus, in comparison to filtering a single individual the inclusion of the parental genotypes reduces the number of variants by a factor of around five. The removal of highly variable genes as observed by the analysis of single individuals cut the remaining variants in half.

**Figure 3:**
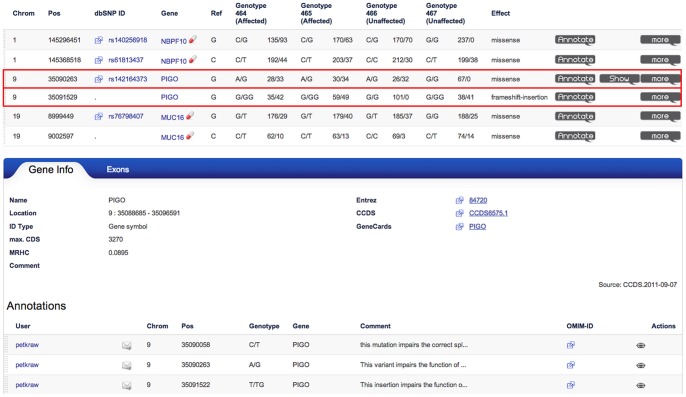
Filtering results for compound heterozyotes in a case study. With the filter settings for genotype frequency <0.01, effect on protein level (functional filter: missense, nonsense, stop loss, splice site, insertions or deletions), and compound heterozygous yields six variants in three genes. *MUC16* and *NBPR10* are both genes from large gene families known for their high variability and detection artifacts due to pseudogenes. The heterozygotes in *PIGO* remain as the likeliest candidates. The *Show* icon at the right end of the line links to an expert curated annotation database that indicates that the mutation in *PIGO* is causing Hyperphosphatasia with mental retardation syndrome and has been published in [9]. The gene view for *PIGO* lists all variant annotations for this gene and links to further knowledge bases. The length of the coding sequence of the longest transcript (max. CDS) and the mean number of rare heterozygous variant calls per exome (MRHC) are important parameters for the assessment of candidate genes.

We continued our analysis with exome data from a family with two daughters affected by Mabry syndrome also known as hyperphosphatasia with mental retardation syndrome (MIM 614749) [9]. The parents were of European descent and unrelated. Altogether 27584 distinct variants were detected in the coding regions of all four analyzed family members. Applying the genotype frequency filter and removing known sequencing artifacts reduced this number to 2208 variants. 1446 of these variants had a predicted effect on the protein level. The compound heterozygous filter reduces this set to six variants in three distinct genes, *NBPF10, MUC16*, and *PIGO* (see Figure 3).

**Figure 4:**
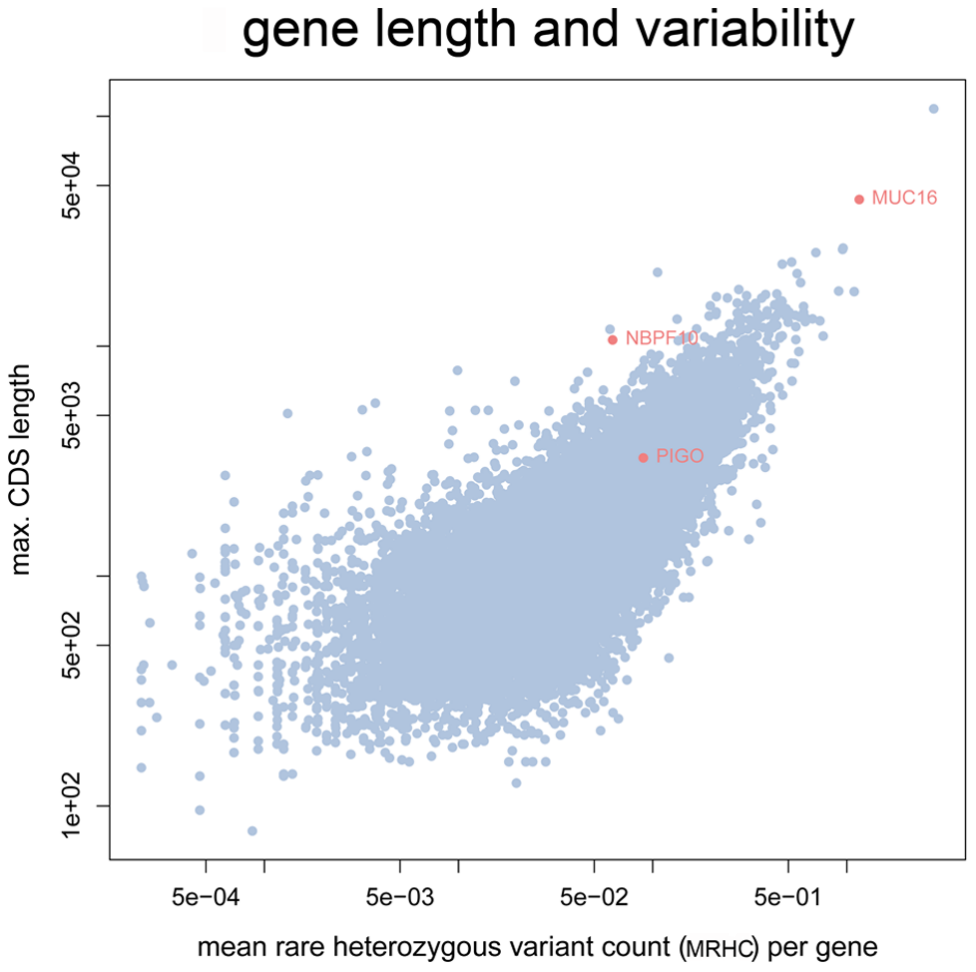
The length of the coding sequence and the mean number of rare alleles per gene. In an average healthy individual from the 5000 exomes project there is more than one rare heterozygous variant in *MUC16* that has an allele frequency below 0.01 in the reference population. In contrast, the coding sequence of *PIGO* is much shorter and rare heterozygous variants occur in less than 8 out of 1000 exomes.

**Figure 5:**
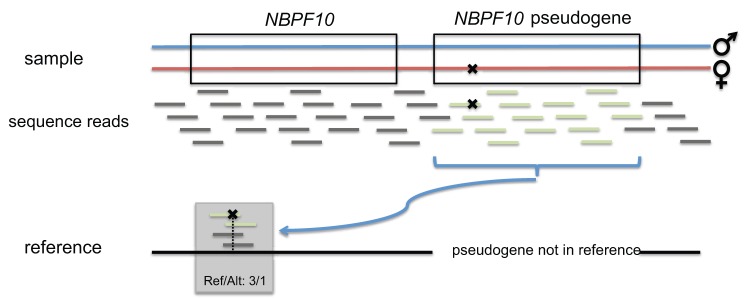
Illustration of mapping artifacts resulting in false positive variant detection. The illustrated sample carries a mutation in the maternal copy of a pseudogene of *NBPF10*. If the pseudogene is not included in the reference sequence, the reads originating from this pseudogene are mismapped. This may result in a false variant call. Indicative for false genotype calls are proportions of reads supporting the alternate allele that strongly deviate from 0.5 or 1.

*MUC16* is a member of a large family of mucin coding genes and *NBPF10* is from the neuroblastoma breakpoint family, which consists of 22 genes and pseudogenes that arose by gene duplication [10], [11]. Both genes are highly variable and harbor many low frequency variants [8]. Genes with a long coding sequence (CDS) and many rare heterozygous variants are more likely to appear as candidate genes after compound heterozygous filtering. In Figure 4 a scatterplot is shown for the mean number of rare heterozygous variants and the CDS length of the longest transcript for each gene. *MUC16* is not only an extraordinarily large gene with over 40kb coding sequence, in average there were also 1.16 rare heterozygous variant calls per healthy individual with an allele frequency below 0.01 in the reference population of the 5000 exomes project. In e.g. NA19238, there were also two such rare heterozygous calls that passed the filter. Genes with pseudogenes, such as *NBPF10* are also prone to genotyping artifacts in reference guided resequencing due to mismapped reads: A variant that is classified as a heterozygous genotype is likely to be a false positive call, if the coverage of this position is high, however the proportion of reads supporting the alternate allele deviates strongly from the value of 0.5 that is expected for a heterozygous genotype. Figure 5 illustrates the mismapping of reads originating from a pseudogene of *NBPF10* that result in such genotyping artifacts. For *NBPF10* many such calling artifacts have been reported in GeneTalk and were automatically excluded. Also the variants detected in *NBPF10* and *MUC16* are highly suggestive for such false calls, for instance in the heterozygously called variant with the dbSNP ID rs61813437 the alternate allele is only supported by 44 out of altogether 236 sequence reads in the first individual and likewise in the others (alternate allele/reference allele: 203/37, 212/30, 199/38) and in dbSNP this variant is also listed as “not validated”. After this quick assessment of the trustworthiness of the variant calls, *PIGO* remains as the most promising disease candidate gene in this case and the mutations were indeed confirmed as the causative mutations of the disorder [9].

With the additional second affected individual in this case scenario the number of candidate genes was markedly lower than in the two trios. In autosomal recessive disorders all affected family members share both haplotypes around the disease locus and are therefore identical by descent for both copies of the gene (IBD = 2) [12]. Two siblings are in average IBD = 2 in only one quarter of their genome which reduces the number of candidate genes likewise with every additional affected sibling. If we analyzed in this family only one of the affected sisters at a time in a trio approach, we would have seen the additional candidate genes *HRNR*, *PLCD1*, and *MUC5B*. KGGseq [13], a statistical framework for analyzing exome data of multiple individuals has only implemented rule 2 for compound heterozygous filtering and reduced to 223 rare candidate variants.

## Conclusions

In this work we developed a filter for identifying compound heterozygotes in exome data of one or more individuals of a family. The rule set on which our filter is based, is comprehensive for analyzing multiple samples and advances the prioritization of compound heterozygous candidate variants beyond existing tools for analyzing data of exome sequencing studies [14], [15]. We showed that filtering for compound heterozygous mutations is an effective means in identifying disease candidate genes especially if multiple family members are available for the analysis. In a trio analysis with exome data of the parents and one affected child, typically, mutations in only about a dozen candidate genes remain. This manageable number of remaining genes can then be assessed based on the expertise of the investigator or further prioritized by suitable tools[16]–[18]. We implemented the compound heterozygous filter as an intuitively usable web service that allows a quick reduction of the exome variants to such a candidate set. The filter as well as the European and Yoruban trios are accessible via the demo account at www.gene-talk.de [1].

### Web Resources

The URL for data presented herein are as follows:

1000 genomes project website. Available: http://www.1000genomes.org. Accessed 2013 May 2.

NHLBI Exome Sequencing Project (ESP) website. Available: http://evs.gs.washington.edu/EVS/. Accessed 2013 May 2.

GeneTalk website. Available: www.gene-talk.de. Accessed 2013 May 2.

## Supporting Information

Table S1 (PDF)
