# Supplementary material for: Filtering for Compound Heterozygous Sequence Variants in Non-Consanguineous Pedigrees
Source: PLoS One. 2013 Aug 5;8(8):e70151. doi: 10.1371/journal.pone.0070151 (PMC3734130; doi:10.1371/journal.pone.0070151)
Supplement: Table S1 — (PDF) [file pone.0070151.s001.pdf]

Gene	pass	Gene	pass	Gene	pass	Gene	pass	Gene	pass	Gene	pass
A2M	2	DDX20	2	LRP1	2	SFI1	2	C15orf2	3	PRR21	3
AARS2	2	DDX27	2	LRP5	2	SH3BGR	2	C9orf131	3	PSG5	3
ABCA3	2	DENND2A	2	LRRC14B	2	SIGLEC1	2	CAPN10	3	PTCD1	3
ACACB	2	DFNB31	2	LRRC56	2	SIX4	2	CARD11	3	PTPRH	3
ACBD4	2	DHX34	2	LRRK1	2	SLC18A1	2	CARD6	3	PTPRZ1	3
ACSL3	2	DHX57	2	LRRN4	2	SLC28A3	2	CCDC110	3	PXDNL	3
ACTRT2	2	DISP2	2	LSR	2	SNX19	2	CD3G	3	RERE	3
ADAM29	2	DLC1	2	MAP1A	2	SPATA16	2	CDKL2	3	RIMS2	3
ADAM32	2	DLEC1	2	MAP7	2	SPATA5L1	2	CDS1	3	RNF207	3
ADCY10	2	DMXL2	2	MCM8	2	SPRED1	2	CEP250	3	ROS1	3
ADCY3	2	DNAH3	2	MICAL1	2	SRFBP1	2	CHI3L1	3	RPAP1	3
ADRM1	2	DNMBP	2	MSS51	2	SSFA2	2	CLTCL1	3	RREB1	3
AGBL5	2	DNPEP	2	MTSS1	2	ST6GALNAC1	2	CNTRL	3	SCAF11	3
AKAP11	2	DOCK6	2	MTUS1	2	ST7L	2	COL16A1	3	SCYL1	3
ALPK3	2	DPP3	2	MYBPC2	2	STEAP1	2	COL2A1	3	SDHA	3
AMZ1	2	DPY19L3	2	MYLK2	2	STON1-GTF2	2	COL3A1	3	SGOL2	3
ANK2	2	DSG3	2	MYO15A	2	STOX1	2	CPNE1	3	SIGLEC12	3
ANKK1	2	ECT2L	2	MYOM3	2	STOX2	2	CPO	3	SLC26A4	3
ANKRD11	2	EHHADH	2	NBAS	2	SUN3	2	CSMD2	3	SLC28A1	3
ANKRD27	2	EIF3C	2	NCAPD3	2	TANC1	2	CSPG4	3	SOGA2	3
ANKRD55	2	EME2	2	NHLRC2	2	TANC2	2	CTNNA3	3	SPATA21	3
ANKZF1	2	EMID1	2	NLRP1	2	TARBP1	2	CYP2A6	3	SRRM2	3
ANO7	2	EP400	2	NOTCH2	2	TBCE	2	DIP2A	3	SSH2	3
AOX1	2	ERCC4	2	NPC1L1	2	TECTA	2	DPY19L2	3	SULT1A1	3
AP5S1	2	ERI1	2	NPNT	2	TENC1	2	DSEL	3	SUMO4	3
APOBEC3H	2	ETAA1	2	NR1H2	2	TEX33	2	DSG2	3	TAF4B	3
ARHGAP32	2	F2RL2	2	NUCB2	2	TFB1M	2	DYNC2H1	3	TAS1R1	3
ARHGAP39	2	FAHD2B	2	NUDT13	2	TGM6	2	F5	3	TCIRG1	3
ARHGEF10	2	FAM176A	2	NUP210L	2	TGOLN2	2	FAM129B	3	TDRD5	3
ARHGEF15	2	FAM65B	2	NUP214	2	THADA	2	FAM149A	3	TEKT5	3
ARHGEF18	2	FAM83H	2	OAS3	2	THOP1	2	FAM188B	3	TEP1	3
ARID5B	2	FANCM	2	OPN1SW	2	THSD7A	2	FAM26E	3	TG	3
ARL11	2	FANK1	2	OR10A5	2	TIAM2	2	FAM43A	3	TLR6	3
ASB16	2	FBXW10	2	OR10J1	2	TJP1	2	FCHO1	3	TMPRSS6	3
ATF3	2	FER1L6	2	OR10K2	2	TLL2	2	FGD5	3	TNFRSF10D	3
ATF7IP2	2	FILIP1L	2	OR1L4	2	TMC3	2	FHOD3	3	TPSD1	3
ATG2A	2	FLNB	2	OR2W3	2	TMCO7	2	FOXRED2	3	TPSG1	3
ATM	2	FLNC	2	OR4M1	2	TMEM184A	2	FUT6	3	TRMT61B	3
ATP6V0A4	2	FMN1	2	OR51F1	2	TMEM198	2	GEMIN4	3	TRPM5	3
ATP8B4	2	FREM1	2	OR51G1	2	TNRC18	2	GEN1	3	TRPM6	3
BAIAP3	2	GAA	2	OR51G2	2	TOP1MT	2	GOLGA4	3	UBR7	3
BAZ1A	2	GBGT1	2	OR51I2	2	TROAP	2	GOLGB1	3	UNC13D	3
BCAR3	2	GBP3	2	OR52H1	2	TRPM4	2	GPR108	3	USP16	3
BCHE	2	GBP4	2	OR5AS1	2	TRPM7	2	GPR179	3	USP32	3
BNC2	2	GGA3	2	OR5H6	2	TTC7A	2	GSG2	3	USP36	3
BOC	2	GGCX	2	OR5R1	2	TTF1	2	GTF2IRD2B	3	VCAN	3

BRD2	2	GPR116	2	OR7C1	2	TTYH2	2	HEATR5B	3	VWF	3
C10orf28	2	GPR128	2	OR8D4	2	TXNDC3	2	HEXDC	3	ZBBX	3
C10orf81	2	GPR158	2	OR8H2	2	UBR4	2	HMMR	3	ZC3HAV1L	3
C12orf35	2	GRB7	2	OR9A2	2	UHRF1BP1L	2	HSD17B4	3	ZCCHC2	3
C12orf40	2	GRHL3	2	OR9G9	2	ULK4	2	IL12RB2	3	ZNF233	3
C15orf27	2	GSTM3	2	OTUD7A	2	UNC13C	2	IL17RC	3	ZNF292	3
C15orf42	2	HEATR1	2	PARP10	2	USH2A	2	JMJD1C	3	ZNF295	3
C16orf46	2	HEATR8	2	PARP3	2	USP29	2	KIAA0415	3	ZNF600	3
C3	2	HECW1	2	PCDHA11	2	USP42	2	KIAA0556	3	ZNF701	3
C8orf74	2	HECW2	2	PCDHB13	2	USP45	2	KLHL38	3	ZNF816	3
C9orf79	2	HSPA1A	2	PCDHGA11	2	UTRN	2	KLK15	3	ZRANB3	3
C9orf96	2	HTR3C	2	PCDHGA2	2	VEZT	2	KRT24	3	ABCA4	4
CACNA2D4	2	HVCN1	2	PCDHGA8	2	WDR52	2	KRT78	3	ABCC3	4
CALCA	2	IDUA	2	PDZRN4	2	WDR63	2	LCE1E	3	AHSA2	4
CAMSAP2	2	IFT172	2	PER2	2	WFS1	2	MACC1	3	ALDH3B2	4
CAPN11	2	IGSF10	2	PET112	2	WNK1	2	MACF1	3	ALPK2	4
CAST	2	IL1RL1	2	PEX5	2	XAF1	2	MAN1B1	3	AQPEP	4
CBX6	2	INADL	2	PGM5	2	XDH	2	METAP2	3	BCAR1	4
CC2D1B	2	INPP5K	2	PIF1	2	YEATS2	2	MIA3	3	C17orf57	4
CCBL2	2	INSL4	2	PITPNM1	2	YSK4	2	MICALL1	3	C1orf101	4
CCDC105	2	IRX6	2	PKD1	2	ZBTB7C	2	MLH3	3	C20orf144	4
CCDC164	2	ISPD	2	PKHD1L1	2	ZC3H13	2	MLPH	3	CALML5	4
CCDC37	2	ITGA7	2	PKMYT1	2	ZFAT	2	MPV17L	3	CASC5	4
CCDC74A	2	ITGB4	2	PLEKHG2	2	ZFPM2	2	MS4A14	3	CCDC40	4
CCDC88B	2	ITPR2	2	PLEKHG5	2	ZFR2	2	MYH1	3	CCDC88C	4
CDC5L	2	KCNK17	2	PLEKHH2	2	ZFYVE19	2	MYH14	3	CEP350	4
CDT1	2	KCNMB3	2	PMFBP1	2	ZNF107	2	MYH4	3	CEP68	4
CELA2A	2	KIAA0182	2	PPP1R3A	2	ZNF16	2	MYH8	3	CGN	4
CENPE	2	KIAA0284	2	PPP6R2	2	ZNF257	2	NCOA4	3	CMYA5	4
CENPJ	2	KIAA0664	2	PRAMEF10	2	ZNF283	2	NEIL3	3	COBL	4
CEP78	2	KIAA0947	2	PRIC285	2	ZNF335	2	NEURL4	3	CORO7-PAM	4
CHTF18	2	KIAA1211	2	PRODH	2	ZNF385D	2	NLRP8	3	CRB2	4
CKAP2	2	KIAA1614	2	PRR5-ARHGA	2	ZNF44	2	NOM1	3	CUBN	4
CLGN	2	KIAA2018	2	PRSS16	2	ZNF77	2	NOMO1	3	DNAH10	4
CLN8	2	KIF16B	2	PTPRN2	2	ZNF804A	2	NOTCH1	3	FAM120A	4
CLUL1	2	KIF24	2	PXK	2	ZNF93	2	NPHS1	3	FAM129A	4
CNGB1	2	KIF7	2	RAB11FIP3	2	ZSCAN5B	2	OR10A4	3	FAM129C	4
COG5	2	KIFC2	2	RADIL	2	AASDH	3	OR1B1	3	FAM193A	4
COL11A1	2	KLHDC4	2	RAET1L	2	ABCC11	3	OR1M1	3	FAM55D	4
COL17A1	2	KLHDC7A	2	RAPGEF6	2	ABCC2	3	OR2T34	3	FANCI	4
COL27A1	2	KNTC1	2	RCSD1	2	ACY3	3	OR6C4	3	GGT1	4
COL28A1	2	KREMEN2	2	RFPL1	2	ADAM2	3	OR8I2	3	GLT6D1	4
COL4A1	2	KRT23	2	RPGRIP1L	2	ADAMTS7	3	PCDHAC1	3	GPR125	4
COL5A1	2	KRT28	2	RPL3L	2	AKAP1	3	PCSK4	3	GPR98	4
CPA5	2	KRT3	2	RPUSD3	2	ALPK1	3	PDILT	3	HHLA2	4
CPNE7	2	KRT35	2	RSL1D1	2	APOBEC3F	3	PDXDC1	3	IGF2R	4
CPS1	2	KRTAP1-5	2	SBNO2	2	ARHGAP11A	3	PIWIL4	3	KANK1	4
CSRP2BP	2	LAMA4	2	SEC14L3	2	ATAD3A	3	PKD1L1	3	KIF26B	4
CTSE	2	LGALS9B	2	SEC16B	2	ATAD3B	3	PKD1L2	3	KLRC3	4
CYP4B1	2	LIG1	2	SEC24D	2	ATAD5	3	PLB1	3	KRT81	4
CYP4F12	2	LILRA3	2	12-Sep	2	ATP8B3	3	PLCE1	3	LCN1	4
DAB2IP	2	LILRB2	2	SERPINA5	2	AXIN2	3	PLXND1	3	LRRC17	4
DCAF4L2	2	LLGL2	2	SERPINA9	2	BRAP	3	PNKP	3	LRRCC1	4
DCLRE1A	2	LRGUK	2	SETD6	2	BSN	3	PPIP5K2	3	MGAM	4

Gene	pass	Gene	pass	Gene	pass	Gene	pass
MLL3	4	TNS1	5	PRDM2	9	AKAP3	43
MYO7B	4	UCMA	5	PRR25	9	OR51A2	44
MYOM2	4	UGGT1	5	TDRD6	9	GLRA3	46
NLRP4	4	ZFYVE28	5	A2ML1	10	CCDC67	48
NUP54	4	ZNF804B	5	AQP12B	10	POTED	48
NVL	4	ZP3	5	HIVEP3	10	ABCA10	49
OR10G7	4	AFF3	6	LRP2	10	CCDC66	51
OR2M5	4	APOB	6	OR13C5	10	C4orf17	52
OR4A16	4	BDP1	6	OR2T35	10	ZBED3	52
OXCT2	4	C1orf173	6	OR2T5	10	ZNF3	52
PCDHB6	4	C2orf16	6	ARHGEF17	11	OR2T4	53
PCNXL3	4	CACNA1H	6	COL4A4	11	PRAMEF22	56
PEBP4	4	CENPF	6	HMHA1	11	CCDC33	62
PIK3C2B	4	CINP	6	HRNR	11	DEFB126	63
PLEKHG1	4	COL22A1	6	MMS22L	11	RIC8A	70
PRAMEF7	4	DHX37	6	OR11G2	11	OR51A4	71
QRICH2	4	DNAH5	6	RHCE	11	PRUNE2	71
RGS22	4	FBLN2	6	USP17L2	11	LENG9	72
RP1	4	FREM2	6	AHNAK	12	TIGD6	80
RYR3	4	KIAA1009	6	CDSN	12	ZNF284	82
SELP	4	KLKB1	6	DHDH	12	AHNAK2	84
SLC38A10	4	KRTAP10-9	6	DNAH7	12	PRICKLE4	86
SLX4	4	LAMA2	6	HMCN1	12	HLA-DRB5	94
SPEN	4	LRRIQ1	6	MDN1	12	HLA-DRB1	99
SPN	4	MKI67	6	OR13C2	12	BCLAF1	163
SPTA1	4	OR2L8	6	OR4D1	12	CDK11B	171
STK33	4	OR2M3	6	TMEM129	12	MUC6	172
TELO2	4	P2RX5	6	ALMS1	13	PDE4DIP	172
TNN	4	PARP4	6	ARHGEF5	13	GPRIN2	173
TPD52L3	4	PCDHB15	6	FBN3	13	KCNJ12	173
TRAPPC9	4	PCDHGA3	6	KIF20B	13	MAP2K3	173
UNC5B	4	PHRF1	6	OR2T27	13	OR4C3	173
VPS13D	4	PLEC	6	PIK3C2G	13		
WDR49	4	PRAMEF2	6	REXO1	13		
WDR90	4	PRPS1L1	6	RYR1	13		
ZNF365	4	PTPN18	6	SYNPO2	13		
ZNF430	4	RASSF7	6	ZNF594	13		
ZNF597	4	RLN1	6	AKAP9	14		
ZNF681	4	TMEM173	6	MPDZ	14		
ZNF789	4	TMPRSS13	6	OR5H14	14		
ZNF831	4	UTP20	6	OR7A10	14		
ZSCAN29	4	XRRA1	6	TEKT4	14		
ADAMTS19	5	ZNF474	6	B4GALNT3	15		
AKAP13	5	ZZEF1	6	CYP3A43	15		
ALLC	5	ACIN1	7	HLA-A	15		
AMBN	5	BCAS1	7	OR1E2	15		

ANK1	5	CA9	7	OR7G3	15		
ANKLE2	5	CLEC4F	7	POM121C	15		
ANKRD26	5	CYP2F1	7	SPEG	15		
AQP12A	5	EPG5	7	KRT33A	16		
ATP10A	5	FAM75A6	7	SYNE1	16		
BAG3	5	GRIN2C	7	KRT77	17		
C2orf71	5	KIAA1683	7	PLIN4	17		
C5	5	KIF26A	7	DEFA4	18		
CSHL1	5	LAMA1	7	LILRA2	18		
CTNNAL1	5	LPA	7	OR51B6	18		
CUL9	5	NEB	7	RSPH10B	18		
DMBT1	5	OR2B11	7	SLC22A1	18		
DNAH2	5	POLE	7	CDHR5	19		
DYSF	5	POLRMT	7	KRT33B	19		
E2F7	5	PPL	7	LAMA5	19		
ESYT3	5	RTKN2	7	CELSR2	20		
FAM179A	5	SH3TC1	7	FLG	20		
FAM82A1	5	USP40	7	COL6A3	21		
FAT4	5	ABCA13	8	GSTM1	21		
GFM2	5	APC	8	LAD1	21		
GPR156	5	CABLES1	8	RP1L1	21		
GZMM	5	COL7A1	8	HLA-C	22		
HELB	5	DCHS2	8	MAVS	22		
HIVEP1	5	DDX54	8	TCP10	22		
HLA-B	5	DNAH1	8	CDRT1	23		
HRG	5	DNAH9	8	FAM75D1	23		
HSPBAP1	5	EVPL	8	GOLGA3	23		
IKBKAP	5	FAT1	8	OR4L1	23		
IQSEC1	5	FLG2	8	PTX4	23		
KIAA1462	5	MST1	8	CEACAM3	24		
LTF	5	MYO5B	8	RHD	24		
MAP3K13	5	NINL	8	ZNF607	24		
MAP4	5	OR5AC2	8	FCGBP	26		
NBPF3	5	PRAMEF1	8	C14orf49	27		
NCAPG2	5	PRAMEF12	8	CDC42EP1	27		
NRAP	5	SACS	8	FYCO1	27		
NUDT17	5	SVEP1	8	OTOP1	27		
OR2T12	5	TMEM82	8	PILRB	28		
OR3A2	5	TRIM16	8	C2CD3	30		
PCIF1	5	TUBGCP6	8	GRIN3B	30		
PCLO	5	XIRP1	8	LILRB4	31		
PEG3	5	ZNF407	8	MAN2A1	31		
PIWIL3	5	AKAP12	9	SERPINB3	31		
PKHD1	5	BRCA2	9	KANK3	32		
PNPLA2	5	CCDC142	9	LILRB1	33		
PPP1R15A	5	CFC1	9	ZFPM1	35		
QRFPR	5	CFC1B	9	ABCA8	37		
REPIN1	5	CR1	9	HLA-DQA1	37		
SOWAHB	5	FAM208B	9	MUC17	38		
SPPL2C	5	FAT2	9	RETNLB	38		
STAB2	5	MICALL2	9	DSG1	40		
STBD1	5	NPHP4	9	FMO2	41		
SYNE2	5	OR2T8	9	RIMBP3B	41		
SYT6	5	PLA2R1	9	WWC1	41		
